# Impact of extreme ambient temperatures on low birth weight: Insights from empirical findings in Pakistan

**DOI:** 10.1177/17455057251341723

**Published:** 2025-06-05

**Authors:** Syed Hira Fatima, Asif Khaliq, Salima Meherali, Zahid Memon, Zohra S. Lassi

**Affiliations:** 1 Global Ecology, Partuyarta Ngadluku Wardli Kuu, College of Science and Engineering, Flinders University, Adelaide, SA, Australia; 2 College of Medicine and Public Health, Flinders University, Adelaide, SA, Australia; 3 School of Public Health, Faculty of Health and Medical Sciences, University of Adelaide, SA, Australia; 4 School of Public Health and Social Work, Queensland University of Technology, Brisbane, Australia; 5 College of Health Sciences, Faculty of Nursing, University of Alberta, Edmonton, Canada; 6 Department of Community Health Sciences, Aga Khan University, Karachi, Pakistan; 7 Robinson Research Institute, Faculty of Health and Medical Sciences, University of Adelaide, SA, Australia

**Keywords:** climate change, maternal and child health, heat, DLNM

## Abstract

**Background:**

Exposure to extreme ambient temperatures during pregnancy, including both heat and cold, can lead to complications such as preterm births, low birth weight (LBW), and developmental anomalies. These exposures pose immediate health risks to both mother and child and may exacerbate health disparities across future generations.

**Objective:**

Pakistan, with limited health resources, is particularly vulnerable to the impacts of extreme temperatures. This study aimed to quantify the association between heat and cold exposure and LBW in Pakistan.

**Design:**

Space-time-series study design.

**Methods:**

We analysed 17,077 birth records from 10 datasets from the Multi-Indicator Cluster Surveys and 1 from the Pakistan Demographic and Health Surveys, covering monthly LBW cases from January 2008 to December 2017. These data were linked to monthly heat index estimates, derived from temperature and humidity, from Copernicus ERA5-Land, aggregated at the provincial level. We used a space-time-series study design with quasi-Poisson distributed lag nonlinear regression. Models were adjusted for long-term trends, seasonality, and socio-economic factors, including maternal education, wealth index and rural residence. We estimated the cumulative risk of LBW associated with heat and cold, individual lag effects and the attributable fraction of LBW cases due to temperature exposure.

**Results:**

LBW was reported in 26.02% (*n* = 4444) of total birth records. The overall exposure–response relationship indicated a positive association between LBW and extreme heat; however, the estimates were imprecise and included the null. At lag 0 (month of conception), there was evidence of increased risk during periods of moderate heat (90th percentile: relative risk (RR) 1.70; 95% confidence interval (CI): 1.01, 2.87) and extreme heat (99th percentile: RR 1.93; 95% CI: 1.00, 3.71). The heat-related attributable fraction for LBW ranged from 0.34 to 0.42 across provinces. In contrast, no association was found between LBW and cold exposure.

**Conclusions:**

This study contributes to the existing body of evidence of the association between extreme temperatures and LBW, particularly from a low-resource, highly vulnerable country. Notably, we found a positive association between heat exposure and LBW during the first month of pregnancy (lag 0), suggesting that early gestation may be a critical period of vulnerability.

## Plain language summary

Extreme temperature exposure and the likelihood of babies being born with low birth weights in Pakistan

**Background:** Exposure to extreme temperatures during pregnancy, whether hot or cold, can lead to serious health problems for babies, such as being born too early, underweight, or with developmental issues. These issues can affect the immediate health of both the mother and child and may contribute to ongoing health disparities in future generations. **Objective:** Pakistan, with its limited healthcare resources, is particularly vulnerable to temperature extremes. This study aims to measure the impact of both heat and cold exposure on low birth weight (LBW) in newborns, using real-world data. **Methods:** We used a method that considered changes over time and differences across various locations. Our analysis included data from 10 surveys conducted in Pakistan, including multiple Multi-Indicator Cluster Surveys (MICS) and one Pakistan Demographic and Health Survey (PDHS), covering the period from January 2008 to December 2017. We linked monthly data on LBW cases from different provinces with weather information, such as temperature and humidity, from a global weather dataset (Copernicus ERA5-Land). **Results:** We found that exposure to extreme temperatures was associated with an increased risk of babies being born underweight. While our results indicated uncertainty, they showed that the first month of pregnancy appeared to be one of the most sensitive periods when temperature exposure could impact birth weight. **Conclusions:** These findings underline the serious impact of extreme temperatures on the health of mothers and babies in Pakistan. They emphasise the importance of policies and programmes to help communities adapt to the effects of climate change. Additionally, our study highlights the need for more comprehensive data collection to strengthen evidence and improve future research.

## Introduction

The escalating threat of climate change poses profound challenges to global health, characterised by rising temperatures and their far-reaching health consequences.^1^ Extreme temperature events—both heat and cold-alongside anthropogenic pollutants and the urban heat island effect—present significant risks to vulnerable populations, particularly those residing in densely populated urban areas with limited green spaces.^2^

Of particular concern are pregnant women, who represent a demographic uniquely susceptible to the adverse effects of extreme temperatures.^3^ The physiological adaptations inherent in pregnancy make women more prone to temperature stress, dehydration and related ailments during extreme heat and cold.^4^ Additionally, pregnant women bear the dual responsibility of safeguarding their health and that of the developing foetus. Consequently, exposure to extreme temperatures during pregnancy can precipitate a spectrum of complications, including but not limited to preterm births, low birth weights (LBW) and developmental anomalies.^4^ Poor pregnancy outcomes not only affect the health and well-being of both mother and child immediately but also have long-lasting repercussions that can impact subsequent generations. These effects can perpetuate cycles of health disparities within affected communities.^5^

Globally, LBW prevalence in low- and middle-income countries (LMICs) is six times higher than in high-income countries, with South Asia contributing to 75% of LBW cases. Despite the severity of these risks, the intricate interplay between climate-induced temperature extremes and adverse pregnancy outcomes remains inadequately elucidated, particularly in LMICs such as Pakistan where the ramifications of climate change are acutely felt.^3,6^ With a large population, rapid urbanisation and limited healthcare resources, Pakistan confronts significant challenges in mitigating the adverse effects of extreme weather events on maternal health. Gender disparities in healthcare access further exacerbate the vulnerability of pregnant women to climate-induced health risks. Alarmingly, neonatal disorders have emerged as the leading cause of death in Pakistan, as highlighted by global estimates from 2019.^7^ The annual death rate for children under-5 stands at 62.9 deaths per 1000 live births, with the corresponding rate of 52.7 deaths per 1000 live births in infants.^8^ Notably, while there has been a decline in these rates over time, specific regions in southern Punjab and northern Baluchistan continue to exhibit a higher risk of child mortality.^9^ Furthermore, Pakistan faces a significant burden of premature births, with an estimated 860,000 occurring annually, resulting in nearly 102,000 child deaths due to related complications. This places Pakistan as the second-ranked country among the top 10 nations contributing to two-thirds of all deaths from preterm birth complications.^10^

Therefore, a comprehensive understanding of these dynamics necessitates an interdisciplinary approach. Through rigorous scientific inquiry, we can discern the underlying mechanisms linking extreme temperature exposure to adverse pregnancy outcomes, thus informing targeted interventions and policy initiatives to improve maternal health outcomes and reduce maternal mortality rates in Pakistan. Despite existing literature’s exploration of the association between extreme heat and pregnancy, the specific implications of extreme temperatures on pregnancy outcomes within the Pakistani context are underexplored.^6^

The primary objective of this study is to quantify the relationship between both heat and cold exposure and the risk of LBW using empirical data from Pakistan. Given Pakistan’s status as one of the most vulnerable countries to the adverse effects of climate change, this research aims to provide critical insights into the specific health risks associated with extreme temperatures in this region.

## Methods

The reporting of this study conforms to the Strengthening the Reporting of Observational Studies in Epidemiology (STROBE) statement.^11^ The STROBE checklist is provided as Supplemental Table S1 to ensure comprehensive and transparent reporting of our study design, data collection, analysis, and results.

### Study area

**Figure 1.**
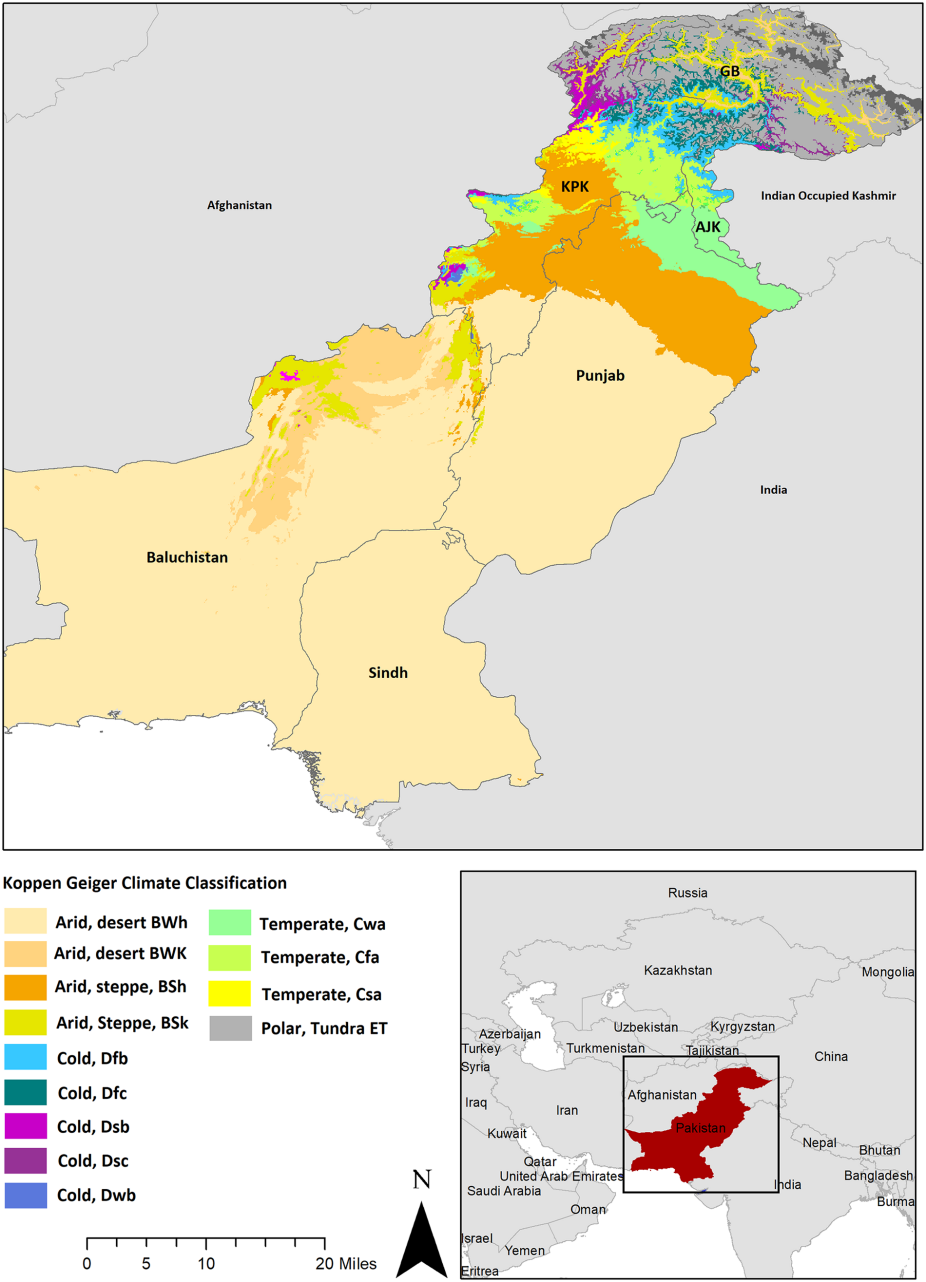
Geographic map illustrating the study area, featuring the location of Pakistan, and overlaid with the Koppen Geiger climate zone^13^ classification for a comprehensive understanding of climate variations across the country.

Pakistan, a South Asian country, is predominantly characterised by a semi-arid climate in the southern regions of Baluchistan and Sindh and a combination of temperate and continental climate in the northern districts in Azad Jammu and Kashmir (AJK), Gilgit Baltistan (GB) and Khyber Pakhtunkhwa (KPK) (Figure 1). With a total population of 224 million and a fertility rate of 3.7, Pakistan falls under the category of LMICs. The country allocates 42 dollars to health per person, and its Universal Health Coverage index is measured at 45 for year 2021.^12^ Administratively Pakistan has 169 districts and 4 provinces (Baluchistan, KPK, Punjab and Sindh), federal territory (Islamabad Capital Territory (ICT)) and 2 autonomous territories (AJK and GB).

### Study design

In this study, we adopted a space-time-series design to examine the association between extreme temperatures and LBW in Pakistan. Retrospective cohort data on mothers and their children were obtained from the Multi-Indicator Cluster Surveys (MICS) and the Pakistan Demographic and Health Survey (PDHS), covering the years 2008 to 2017. This analysis used area-level aggregated data, where monthly LBW cases and exposure variables were summarised at the provincial level to capture broader temporal and spatial trends. This approach aligns with earlier work that applied administrative-level data aggregation in climate-health research.^14^

Data were aggregated at a monthly level to address limitations in the MICS and PDHS datasets, where the exact day of birth was unavailable. Month and year were used as indicators for each child’s birth month.^15^ This design allowed us to assess long-term monthly variations in LBW associated with extreme temperatures, capturing trends over time.^16^ Furthermore, our spatial unit of analysis was at the provincial level. Data were aggregated at this level because precise geographic points were not available in the MICS and PDHS datasets, limiting our ability to align individual data with specific exposure locations.^17,18^ Provincial-level analysis offered sufficient statistical power by capturing broader spatial patterns. Conducting the analysis at the district level would likely introduce many zero counts due to small sample sizes within districts, potentially compromising the robustness of the models.^19^ Aggregating data at the monthly and provincial levels allowed us to capture both temporal and spatial variations in LBW associated with extreme temperatures across Pakistan.^20^

### Datasets

#### Health datasets

We gathered LBW data by consolidating information from 11 datasets obtained from the MICS and 1 from the Pakistan Demographic Health Survey (PDHS, 2012–2013). The MICS datasets included two from MICS-4 (2010–2011), four from MICS-5 (2014–2017) and four from MICS-6 (2017–2020),^17,18^ while the PDHS data came from the 2012–2013 survey. Data from the PDHS 2017–2018 survey were excluded due to potential overlap with the 2018 MICS survey. These surveys provided data for four provinces of Pakistan, as well as from ICT and GB. Due to the small number of cases and geographic proximity, data from ICT were merged with that from Punjab. This combined dataset offers a nationally representative sample of maternal and child health, collected from married women of reproductive age (15–45 years) in Pakistan.^21^

The surveys provided detailed individual-level information on participants’ health, demographics and socio-economic status, which was used to create area-level aggregated measures for analysis. Our analysis focused on mothers who had given birth within the last 5 years, with birth weight information obtained through survey data between 2008 and 2017. We included all cases with LBW data, specifically when birth weights recorded were 2.5 kg or less. In total, we aggregated data from 15,247 records from the MICS datasets and 1830 records from the PDHS dataset, covering the period from 2008 to 2017. The cases were then aggregated by province and month and year of each child’s birth.

#### Climatic datasets

We obtained gridded monthly meteorological datasets from Copernicus ERA5-Land, which include mean temperature (°C), dew point (°C) and precipitation (m).^22^ These datasets have a spatial resolution of 9 × 9 km. Relative humidity (%) was estimated using mean temperature and dew point, and the heat index was calculated using the weathermetrics package in R. This package implements the formulation developed by Anderson et al.,^23^ which incorporates a standard empirical algorithm to compute the heat index based on mean temperature and relative humidity. The data extraction process utilised centroid points for all districts in Pakistan. We then estimated the monthly average of climatic variables within each province to capture local variations. Finally, the monthly climatic data was combined with the monthly LBW cases based on month, year and province.

### Statistical analysis

This study assessed the nonlinear and lagged relationship between exposure to extreme ambient temperatures,^24^ represented by the monthly mean heat index and the risk of LBW in Pakistan. Existing evidence suggests that temperature-related variations in birth weight by gestational age may not be immediately apparent over short time intervals. Monthly exposure assessments, therefore, provide a more refined approach to capturing the potential susceptibility to heat exposure during pregnancy.^25^

We employed a space-time-series study design using distributed lag nonlinear models (DLNMs) within a generalised linear model (GLM) framework, suitable for aggregated count data.^14,26^ This approach allowed us to account for both spatial heterogeneity across provinces and temporal variations over time, while simultaneously estimating the delayed and nonlinear relationship of heat exposure and LBW risk.^14^

Given the count nature of our aggregated data and the presence of overdispersion, we used a quasi-Poisson distribution. The population at risk, represented by the number of women of reproductive age, was included as an offset term to adjust for population size differences across provinces.

The cross-basis function was constructed to model the nonlinear and lagged association between the heat index and LBW. The heat index was modelled using a natural cubic spline with an internal knot placed at the 50th percentile to capture potential nonlinearity. The lag dimension (representing the delayed effect of exposure) was modelled with a natural cubic spline with 3 degrees of freedom (df) over a maximum lag period of 9 months, representing the entire pregnancy period, with lag 0 corresponding to the month of birth.^14^ This configuration allowed us to estimate the cumulative association between heat index exposure from conception to delivery and LBW, accounting for the possibility that heat exposure at different stages of pregnancy might have varying effects on birth outcomes.^14^

To adjust for long-term temporal trends and seasonal variation, we included: (1) A natural cubic spline function of time (date) with 15 df to capture smooth, nonlinear long-term trends. (2) An indicator variable for month of the year to control for residual seasonality. Several socio-economic variables were included to account for potential confounding including: (1) Maternal education: Proportion of mothers with no or low education. (2) Wealth index: Proportion of mothers in the poorer/poorest wealth categories. (3) Rural proportion: Proportion of rural residents within each province-month aggregation.

These area-level covariates were estimated as proportions to reflect population-level socio-economic context. Socio-economic factors are well-established determinants of LBW, influencing maternal health, access to healthcare and overall living conditions.^27^ Missing values in these covariates were addressed using multiple imputation (mice), with predictive mean matching for continuous variables and polytomous regression for categorical variables. Imputation was performed using the MICE package in R.^28^

The primary model was estimated using quasi-Poisson GLM, with relative risks (RRs) and 95% confidence intervals (CI) calculated for the 1st percentile (extreme cold), 10th percentile (moderate cold), 90th percentile (moderate heat) and 99th percentile (extreme heat) of heat index, relative to the province-specific median heat index.^24,28^ The median heat index was identified as the temperature associated with the minimum risk of LBW.^26,29^

Attributable fractions (AF) and attributable numbers (AN) for both heat and cold were estimated using pooled RR estimates with empirical confidence intervals at the provincial level. Heat exposure was defined as monthly heat index values above the province-specific median heat index, and cold exposure as values below the median. The AF and AN were estimated using the following standard formula:

$$\mathrm{AF}=\frac{\left(\mathrm{RR}-1\right)}{\mathrm{RR}}$$

AN=AF×cases

where RR represents the relative risk associated with heat index values and *cases* represent the total LBW cases. To quantify uncertainty, we conducted Monte Carlo simulations (*n* = 10,000) by sampling from the estimated log-normal distribution of the RRs to obtain 95% empirical CI for AF.

Sensitivity analysis was conducted to assess model performance and optimise modelling parameters. We assessed the degrees of freedom for the spline function representing the long-term time trend, testing values between 3 and 30 across the study period, guided by previous studies recommending flexible splines for long-term temporal trends.^29,30^ We also tested different maximum lag periods, comparing 7, 8 and 9 months, to identify the optimal lag period for capturing delayed effects. The selection of degrees of freedom for the heat index predictor spline, long-term time trends, and lag-response function was based on minimising the quasi Akaike information criterion (QAIC) across different combinations. Additionally, we compared quasi-Poisson GLM models to generalised mixed linear models (GLMM) with random intercepts and zero inflation fitted using the glmmTMB package, with the latter accounting for potential unobserved heterogeneity, overdispersion using negative binomial regression and zero inflation. Model configurations were assessed using QAIC and 95% CI to identify the best-performing model. All data preprocessing and statistical analyses were conducted in R (version 4.1.0) using the dlnm,^31^ glmmTMB^32^ and splines packages.^33^

## Results

### Descriptive statistics

After data cleaning and preprocessing, we retained 17,077 records containing birth weight information for the most recent live births reported by each mother between 1 January 2008 and 31 December 2017. Within this cohort, 26.02% of children were classified as having LBW. The distribution varied across provinces, with the highest proportion of cases reported from Punjab (66.7%). Descriptive statistics for LBW cases and environmental variables are presented in Table 1. A consistent seasonal and temporal pattern was observed, with the number of LBW cases increasing over time and peaking between June and September, although some data gaps were present (Supplemental Figure S1).

**Table 1.** Descriptive statistics of LBW cases and environmental conditions in Pakistan from 2008 to 2017 (*N* = 17,077).

Variable	Value (percentage/range)
Total LBW cases	4444 (26.02%)
Baluchistan	68 (1.5%)
GB	225 (5.0%)
KPK	405 (9.0%)
Punjab	2964 (66.7%)
Sindh	782 (17.6%)
Monthly LBW cases,^a^ mean (range)	12.18 (1–78)
Environmental variables, mean (range)	
Heat index (°C)	18.11 (−20 to 38.97)
Temperature (°C)	17.74 (−20.02 to 35.12)
Relative humidity (%)	53.81 (18.93 to 80.19)

LBW: low birth weight; GB: Gilgit Baltistan; KPK: Khyber Pakhtunkhwa.

a Mean number of LBW cases recorded per province-month over the study period (2008–2017).

### Risk estimates of LBW associated with temperature conditions

**Figure 2.**
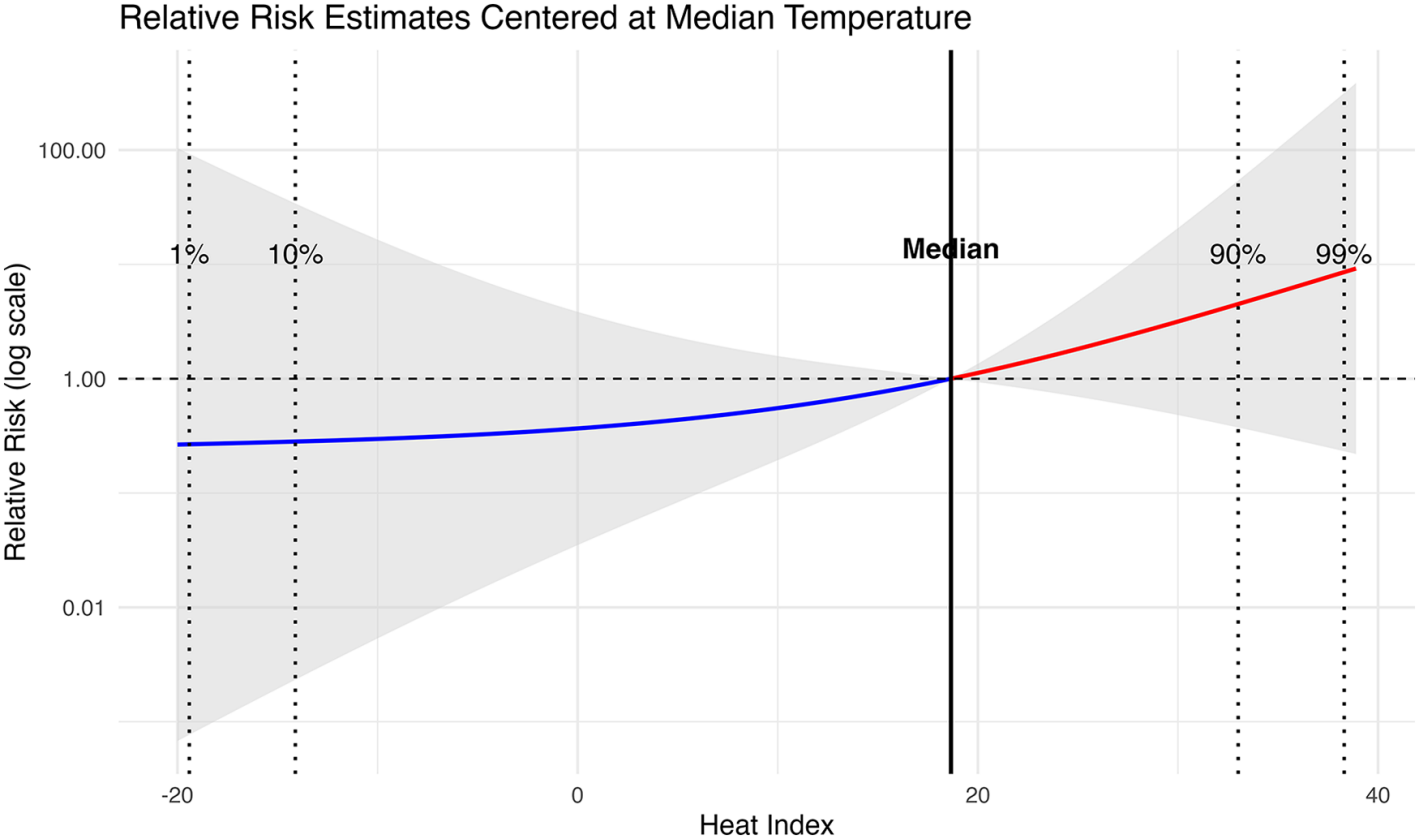
Cumulative exposure–response association between LBW and heat index. Dotted lines represent temperature extremes at both moderate and extreme conditions (hot and cold). The solid vertical line represent median heat index used as a reference value. Dotted vertical lines indicate percentiles corresponding to temperature extremes (1st, 10th, 90th and 99th percentiles). The shaded grey area represents the 95% confidence interval around the relative risk estimates. The *y*-axis is presented on a logarithmic scale for improved visual interpretation of risk estimates across the exposure range. LBW: low birth weight.

Our results suggest a potential positive association between heat exposure and LBW; however, the effect estimates are highly uncertain, with wide confidence intervals that include the null. The cumulative exposure–response relationship over a lag period of 0–9 months between the heat index and LBW risk is presented in Figure 2 and summarised in Supplemental Table S2. Compared to the median heat index (18.6°C), the cumulative RR of LBW was 5.58 (95% CI: 0.32, 97.31) at moderate heat (90th percentile, 34.7°C) and 8.55 (95% CI: 0.23, 314.53) at extreme heat (99th percentile, 38.3°C). Subnational analyses revealed similar trends across provinces, with cumulative RRs at extreme heat ranging from 5.40 in Sindh to 12.08 in KPK. However, all estimates were highly uncertain, with wide confidence intervals that included the null value. In contrast, cumulative RRs for cold exposure (1st and 10th percentiles) showed no consistent association with LBW across provinces, and the estimates similarly had wide confidence intervals that included the null.

With regard to individual lagged effects, we observed a positive association primarily between lag 0 (month of conception) and LBW risk. Specifically, exposure to moderate heat (90th percentile) during the month of conception was associated with a 70% increase in the risk of LBW (RR: 1.70; 95% CI: 1.01, 2.87), while exposure to extreme heat (99th percentile) was associated with a 93% increase in risk (RR: 1.93; 95% CI: 1.00, 3.71) (Table 2). Lag effects beyond the month of conception were imprecise, with confidence intervals including the null, and should be interpreted with caution. No evidence of association was observed between cold exposure and LBW across any lag period.

**Table 2.** The estimated monthly RR and 95% confidence intervals of LBW at moderate heat and extreme heat compared to the reference heat index across different lag months in Pakistan.

Lag months	Risk at extreme cold (1st percentile)	Risk at moderate cold (10th percentile)	Risk at moderate heat (90th percentile)	Risk at extreme heat (99th percentile)
0	0.50 (0.16, 1.58)	0.60 (0.29, 1.22)	1.70 (1.01, 2.87)	1.93 (1.00, 3.71)
1	0.67 (0.27, 1.63)	0.73 (0.42, 1.27)	1.46 (0.96, 2.20)	1.59 (0.95, 2.66)
2	0.87 (0.43, 1.79)	0.88 (0.56, 1.37)	1.26 (0.90, 1.77)	1.34 (0.88, 2.04)
3	1.08 (0.57, 2.07)	1.02 (0.68, 1.53)	1.12 (0.82, 1.53)	1.16 (0.79, 1.70)
4	1.23 (0.66, 2.32)	1.12 (0.74, 1.67)	1.03 (0.76, 1.40)	1.04 (0.72, 1.52)
5	1.26 (0.70, 2.29)	1.14 (0.78, 1.67)	1.00 (0.75, 1.33)	1.00 (0.70, 1.43)
6	1.16 (0.69, 1.96)	1.08 (0.78, 1.50)	1.01 (0.77, 1.32)	1.02 (0.73, 1.42)
7	0.98 (0.61, 1.59)	0.98 (0.74, 1.29)	1.06 (0.81, 1.39)	1.08 (0.77, 1.52)
8	0.79 (0.45, 1.39)	0.85 (0.61, 1.17)	1.15 (0.82, 1.60)	1.18 (0.78, 1.80)
9	0.62 (0.28, 1.34)	0.72 (0.46, 1.14)	1.25 (0.81, 1.94)	1.32 (0.75, 2.29)

LBW: low birth weight; RR: relative risk.

### Attributable fraction

AF estimates suggested that heat exposure contributed to LBW cases across all provinces, with AFs ranging from 0.38 (95% CI: −0.62, 0.74) in Baluchistan to 0.42 (95% CI: –0.60, 0.75) in KPK, though the confidence intervals were wide and included the null value (Table 3). The estimated number of heat-attributable LBW cases varied from approximately 27 cases in Baluchistan to 1082 cases in Punjab.

**Table 3.** Province-specific heat- and cold-related AF and AN of LBW cases with 95% empirical confidence intervals. Heat and cold were defined based on monthly heat index values above and below the province-specific median, respectively.

Provinces	AF in cold	AN	AF in heat	AN
Baluchistan	−50.42 (−229.7, −0.26)	—	0.38 (−0.62, 0.74)	26.85
GB	—	—	—	—
KPK	−15.88 (−91.84, −0.16)	—	0.42 (−0.60, 0.75)	169.74
Punjab	−156.38 (−618.71, 0.11)	—	0.34 (−3.28, 0.99)	1019.45
Sindh	−282.57 (−1162.68, 0.03)	—	0.37 (−0.39, 0.75)	288.10

GB: Gilgit Baltistan; KPK: Khyber Pakhtunkhwa; AF: attributable fraction; AN: attributable number.

In contrast, AF estimates for cold exposure were highly uncertain, with confidence intervals spanning negative values, indicating substantial uncertainty and the possibility of no association or even a protective relationship.

We conducted an in-depth sensitivity analysis to optimise model specifications and inform the selection of key parameters (Supplemental Table S3). Across all models, estimates remained highly uncertain. Models with 15–20 df for long-term trends tended to provide a better fit, whereas lower df values (e.g. 3) appeared to result in overestimation. We also tested maximum lag periods of 7, 8 and 9 months to account for delayed associations across the pregnancy period, with a 9-month lag showing slightly improved model fit. Additionally, a comparison between the two modelling approaches (GLM versus GLMM) indicated a consistently lower QAIC for the GLM approach.

## Discussions

This study represents the first attempt to quantify the association between extreme temperatures and LBW nationally in Pakistan, examining the nonlinear and lagged associations between heat exposure and LBW. LBW poses a significant risk to neonatal morbidity, contributing to long-term health challenges. Our analysis suggests that over 26.02% of babies were reported to be born with LBW, a figure slightly higher than previously reported estimates from Pakistan.^34,35^ We observed an overall positive association between LBW and heat in Pakistan; however, the estimates were highly imprecise and included the null value. It is essential to note that these results come with inherent uncertainties potentially due to the relatively small sample size and limitations in data quality. These factors contribute to wide confidence intervals and high variability in our estimates, underscoring the need for caution when interpreting the findings. Future investigations with more extensive data collection and improved methodologies are warranted to substantiate these initial observations. Despite these limitations, the observed positive association in the month of conception (lag 0) highlights the potential vulnerability of the population and the need for adaptive measures to mitigate health risks during extreme weather events, especially in the context of climate change.

Only recently, there has been widespread recognition that pregnant women are especially susceptible to the negative effects of heat stress.^6^ Pregnancy increases women’s susceptibility to heat exposure, due to physiological and anatomical changes. Internal heat production rises with foetal and placental metabolism, increased body mass and physical strain, posing challenges to thermoregulation.^36–38^ Additionally, social vulnerabilities emerge, as pregnant women in developing countries, including poorer women, may continue to engage in strenuous activities to support their households, potentially exceeding heat tolerance thresholds and endangering their health.^39^

Our study suggests a possible positive association between extreme heat exposure and LBW, aligning directionally with previous studies conducted in other settings. For instance, a recent review of 51 studies found that most reported an increased risk of LBW associated with heat exposure.^40^ Other studies have also reported associations between heat exposure and LBW, supporting the broader evidence that elevated temperatures may pose risks to foetal development.^41–43^ However, differences in study design, exposure definitions and population characteristics limit the comparability of effect estimates across studies. For example, while one study assessed trimester-specific exposures using daily temperature bins,^41^ our study used monthly average heat index values and examined individual months across the pregnancy period, including the month of conception (lag 0). Additionally, our effect estimates are highly uncertain and imprecise, with confidence intervals that include the null. These findings should therefore be interpreted with caution and underscore the need for further research in diverse settings.

We observed an elevated risk of LBW associated with heat exposure during the first month of pregnancy (lag 0), with risk estimates suggesting a 70%–93% increase under hot weather conditions. These findings indicate that early pregnancy, particularly the period around conception, may represent a potentially sensitive period for heat exposure. Heat-related stress during this period could disrupt placental development or impair early foetal growth, thereby contributing to an increased risk of adverse birth outcomes such as LBW. Extreme heat may also exacerbate maternal physiological stress, alter cardiovascular function or reduce nutrient delivery to the developing foetus, potentially compromising foetal development. Our results are broadly consistent with findings from a study in Thatta, Pakistan, which reported an association between heat exposure during the first trimester and increased LBW risk,^3^ reinforcing the importance of early gestational periods as a potential window of vulnerability.

Previous studies suggest that exposure to cold increase the risk of LBW in southern Asia and parts of Africa.^44^ However, we did not observe a clear association between cold exposure and LBW in our analysis. Localised investigations—for example in Baluchistan, KPK, and GB—may reveal important patterns, as high land regions in Baluchistan and KPK have continental semi-arid Mediterranean climate with hot and dry summers and very cold winters and GB characterised by cold desert-climate, situated within the Himalayan and Karakoram Mountain ranges. The remoteness and geographic isolation of some of these regions likely contributed to a smaller sample size in our dataset, limiting the precision of estimates and our ability to draw robust conclusions for this area. Future research should prioritise remote and resource-limited regions, employing locally tailored data collection efforts to better capture the potential health associations of cold exposure in these vulnerable settings.

Geographically, temperature-related AFs were similar across provinces in Pakistan, ranging from 34% to 42%, but with high uncertainty and confidence intervals including the null. There is a need for deeper understanding of covarying climatic and socio-economic factors, such as socio-economic status and access to healthcare facilities, that may contribute to these trends. Further investigations at the local level are warranted to understand these patterns, identify high-risk areas and enhance policy implementation for risk prevention.

Our findings are concerning within the framework of climate change scenarios. The combined effects of rising temperatures and frequent extreme weather events, including heatwaves, cold spells, floods and droughts, will have substantial implications for maternal and child health. This challenge is particularly pronounced in countries like Pakistan, where a multitude of issues, including poor maternal and child health, political and economic instability and the adverse effects of climate change, intersect.^45^ To formulate effective policies, urgent attention is required for evidence-based studies that delve into the intricate relationship between shifting climate patterns and their impacts on maternal health.

In terms of interventions, it is essential to develop a multifaceted approach, to enhance maternal and child health outcomes during periods of both extreme heat and cold. Healthcare providers should be equipped with guidelines focusing on the early identification and prevention of heat stress in pregnant women.^46^ Educational programmes for pregnant women are vital, providing information on recognising heat stress symptoms and adopting preventive measures.^47^ Community outreach efforts, in collaboration with local organisations, should be implemented to raise awareness and offer support to pregnant women. Additionally, advocating for workplace policies that protect pregnant women from excessive heat exposure is crucial. This includes provisions for breaks, hydration access and flexible work schedules during heatwaves. The integration of heat stress considerations into existing maternal and child health policies, alongside telehealth services for remote consultations, can ensure timely interventions without unnecessary risks. Supporting research initiatives and establishing surveillance systems will further deepen our understanding of the specific risks and consequences, facilitating evidence-based approaches to safeguard the health of pregnant women and newborns during extreme heat events.^48^

### Strengths and limitations

1. Analysis at the monthly level: Due to the substantial proportion of missing data on the exact day of birth, we conducted the analysis at the monthly level. This approach allowed us to capture broader, long-term patterns of temperature exposure on LBW risk.
2. Data aggregation and quality considerations: Due to the absence of geospatial data (e.g. Global Positioning System (GPS) coordinates), health and exposure data were aggregated at the provincial and monthly levels. This pragmatic approach helped address data sparsity, which would have been more pronounced at finer spatial scales (e.g. district level). However, survey-based datasets such as DHS and MICS are inherently subject to variability, recall bias and data gaps, particularly in resource-limited settings. This highlights the pressing need for more comprehensive and high-quality data collection in LMICs like Pakistan. The substantial burden of LBW in these regions, combined with data limitations, underscores the importance of better-resourced studies to enable a more accurate and complete understanding of these health risks.
3. Ecological fallacy and exposure misclassification: This study’s reliance on aggregated data at the provincial and monthly scale, necessitated by the unavailability of exact birth dates and GPS coordinates in survey datasets, introduced limitations related to exposure misclassification and the ecological fallacy. Assigning province-level monthly average temperature data as a proxy for individual exposure likely resulted in exposure measurement error, as it cannot account for micro-scale spatial and temporal variations in maternal heat exposure during pregnancy. Furthermore, the aggregation of health outcomes limits our ability to adjust for individual-level confounders, such as maternal health status, occupation and household-level characteristics, which may influence both maternal heat exposure and LBW risk.

These limitations highlight the potential for ecological fallacy, where associations observed at the group level may not reflect individual-level relationships. This warrants caution in interpreting our findings as causal. Future research with individual-level exposure data, precise birth timing and spatially resolved temperature measurements would enhance the accuracy and validity of temperature-LBW risk estimates in this region.

Despite these limitations, this study makes an important contribution to the existing literature from LMICs in the South Asia region, which is particularly vulnerable to the impacts of climate change. It underscores the pressing need for more comprehensive and granular data collection to better inform public health strategies in these high-burden settings.

## Conclusions

This study provides preliminary insights into the potential associations between extreme temperatures and LBW in Pakistan, highlighting the nonlinear and lagged associations. Notably, we found a strong positive association between heat exposure and LBW during the first month of pregnancy (lag 0), suggesting that early gestation may be a critical period of vulnerability. However, estimates beyond this period were more variable and imprecise, reflecting the inherent limitations of survey-based data, particularly in resource-limited settings where data availability can be sparse and inconsistent. The observed variability across model specifications underscores the need for cautious interpretation.

Further research is needed to address data gaps and enhance the reliability and precision of estimates in low-resource developing countries like Pakistan. Strengthening routine data collection systems and integrating higher-resolution health and environmental data will be crucial for improving our understanding of temperature-related risks to maternal and child health. Location-specific evidence is essential for identifying high-risk areas and informing targeted public health interventions and adaptation strategies in these regions amid the challenges of climate change.

## Supplemental Material

sj-docx-1-whe-10.1177_17455057251341723 – Supplemental material for Impact of extreme ambient temperatures on low birth weight: Insights from empirical findings in Pakistan Supplemental material, sj-docx-1-whe-10.1177_17455057251341723 for Impact of extreme ambient temperatures on low birth weight: Insights from empirical findings in Pakistan by Syed Hira Fatima, Asif Khaliq, Salima Meherali, Zahid Memon and Zohra S. Lassi in Women's Health
