# Supplementary material for: Impact of extreme ambient temperatures on low birth weight: Insights from empirical findings in Pakistan
Source: Womens Health (Lond). 2025 Jun 5;21:17455057251341723. doi: 10.1177/17455057251341723 (PMC12144394; doi:10.1177/17455057251341723)
Supplement: sj-docx-1-whe-10.1177_17455057251341723 – Supplemental material for Impact of extreme ambient temperatures on low birth weight: Insights from empirical findings in Pakistan [file sj-docx-1-whe-10.1177_17455057251341723.docx]

**Impact of Extreme Ambient Temperatures on Low Birth Weight: Insights from Empirical Findings in Pakistan**

Syeda Hira Fatima^1,2,3^, Asif Khaliq^4^, Salima Meherali^5^, Zahid Memon^6^, Zohra S Lassi^3,7^*

1. College of Science and Engineering, Flinders University, Australia
2. College of Medicine and Public Health, Flinders University, Australia
3. School of Public Health, Faculty of Health and Medical Sciences, University of Adelaide, Australia
4. School of Public Health and Social Work, Queensland University of Technology, Brisbane, Australia.
5. College of Health Sciences, Faculty of Nursing, University of Alberta, Canada
6. Department of Community Health Sciences, Aga Khan University, Pakistan.
7. Robinson Research Institute, Faculty of Health and Medical Sciences, University of Adelaide, Australia

*Corresponding Author

School of Public Health, Faculty of Health and Medical Sciences, University of Adelaide, Australia

Level 4, School of Public Health, 50 Rundle Mall Plaza, Rundle Mall, Adelaide – 5000

[zohra.lassi@adelaide.edu.au](mailto:zohra.lassi@adelaide.edu.au)

**Supplementary Materials**

**Table of contents**

Table S1 STROBE Statement—Checklist of items that should be included in reports of *cohort studies*

Figure S1 Monthly distribution and patterns of LBW cases from 2008 to 2017.

Table S2 Cumulative and province-specific risk estimates of low birth weight (LBW) associated with heat index exposure.

Table S3 Sensitivity analysis of model configurations assessing the relationship between temperature extremes and low birth weight (LBW).

**Table S1 STROBE Statement—Checklist of items that should be included in reports of *cohort studies***

|  | Item No | Recommendation | Page No |
| --- | --- | --- | --- |
| **Title and abstract** | 1 | (*a*) Indicate the study’s design with a commonly used term in the title or the abstract | 1 |
|  |  | (*b*) Provide in the abstract an informative and balanced summary of what was done and what was found | 1 |
| Introduction | | | |
| Background/rationale | 2 | Explain the scientific background and rationale for the investigation being reported | 2 |
| Objectives | 3 | State specific objectives, including any prespecified hypotheses | 2 |
| Methods | | | |
| Study design | 4 | Present key elements of study design early in the paper | 3 |
| Setting | 5 | Describe the setting, locations, and relevant dates, including periods of recruitment, exposure, follow-up, and data collection | 3 |
| Participants | 6 | (*a*) Give the eligibility criteria, and the sources and methods of selection of participants. Describe methods of follow-up | 3 |
|  |  | (*b*) For matched studies, give matching criteria and number of exposed and unexposed | N/A |
| Variables | 7 | Clearly define all outcomes, exposures, predictors, potential confounders, and effect modifiers. Give diagnostic criteria, if applicable | 3 |
| Data sources/ measurement | 8* | For each variable of interest, give sources of data and details of methods of assessment (measurement). Describe comparability of assessment methods if there is more than one group | 3 |
| Bias | 9 | Describe any efforts to address potential sources of bias | 3 |
| Study size | 10 | Explain how the study size was arrived at | 3 |
| Quantitative variables | 11 | Explain how quantitative variables were handled in the analyses. If applicable, describe which groupings were chosen and why | 3 |
| Statistical methods | 12 | (*a*) Describe all statistical methods, including those used to control for confounding | 4 |
|  |  | (*b*) Describe any methods used to examine subgroups and interactions | N/A |
|  |  | (*c*) Explain how missing data were addressed | 4 |
|  |  | (*d*) If applicable, explain how loss to follow-up was addressed | N/A |
|  |  | (*e*) Describe any sensitivity analyses | 5 |
| Results | | |  |
| Participants | 13* | (a) Report numbers of individuals at each stage of study—eg numbers potentially eligible, examined for eligibility, confirmed eligible, included in the study, completing follow-up, and analysed | 4 |
|  |  | (b) Give reasons for non-participation at each stage | N/A |
|  |  | (c) Consider use of a flow diagram | N/A |
| Descriptive data | 14* | (a) Give characteristics of study participants (eg demographic, clinical, social) and information on exposures and potential confounders | 6 |
|  |  | (b) Indicate number of participants with missing data for each variable of interest | N/A |
|  |  | (c) Summarise follow-up time (eg, average and total amount) |  |
| Outcome data | 15* | Report numbers of outcome events or summary measures over time | 5 |

| Main results | 16 | (*a*) Give unadjusted estimates and, if applicable, confounder-adjusted estimates and their precision (eg, 95% confidence interval). Make clear which confounders were adjusted for and why they were included | 6 |
| --- | --- | --- | --- |
|  |  | (*b*) Report category boundaries when continuous variables were categorized | N/A |
|  |  | (*c*) If relevant, consider translating estimates of relative risk into absolute risk for a meaningful time period | 6 |
| Other analyses | 17 | Report other analyses done—eg analyses of subgroups and interactions, and sensitivity analyses | 6 |
| Discussion | | | |
| Key results | 18 | Summarise key results with reference to study objectives | 6 |
| Limitations | 19 | Discuss limitations of the study, taking into account sources of potential bias or imprecision. Discuss both direction and magnitude of any potential bias | 7 |
| Interpretation | 20 | Give a cautious overall interpretation of results considering objectives, limitations, multiplicity of analyses, results from similar studies, and other relevant evidence | 7 |
| Generalisability | 21 | Discuss the generalisability (external validity) of the study results | 7-8 |
| Other information | | | |
| Funding | 22 | Give the source of funding and the role of the funders for the present study and, if applicable, for the original study on which the present article is based | 9 |

*Give information separately for exposed and unexposed groups.

**Note:** An Explanation and Elaboration article discusses each checklist item and gives methodological background and published examples of transparent reporting. The STROBE checklist is best used in conjunction with this article (freely available on the Web sites of PLoS Medicine at http://www.plosmedicine.org/, Annals of Internal Medicine at http://www.annals.org/, and Epidemiology at http://www.epidem.com/). Information on the STROBE Initiative is available at http://www.strobe-statement.org.


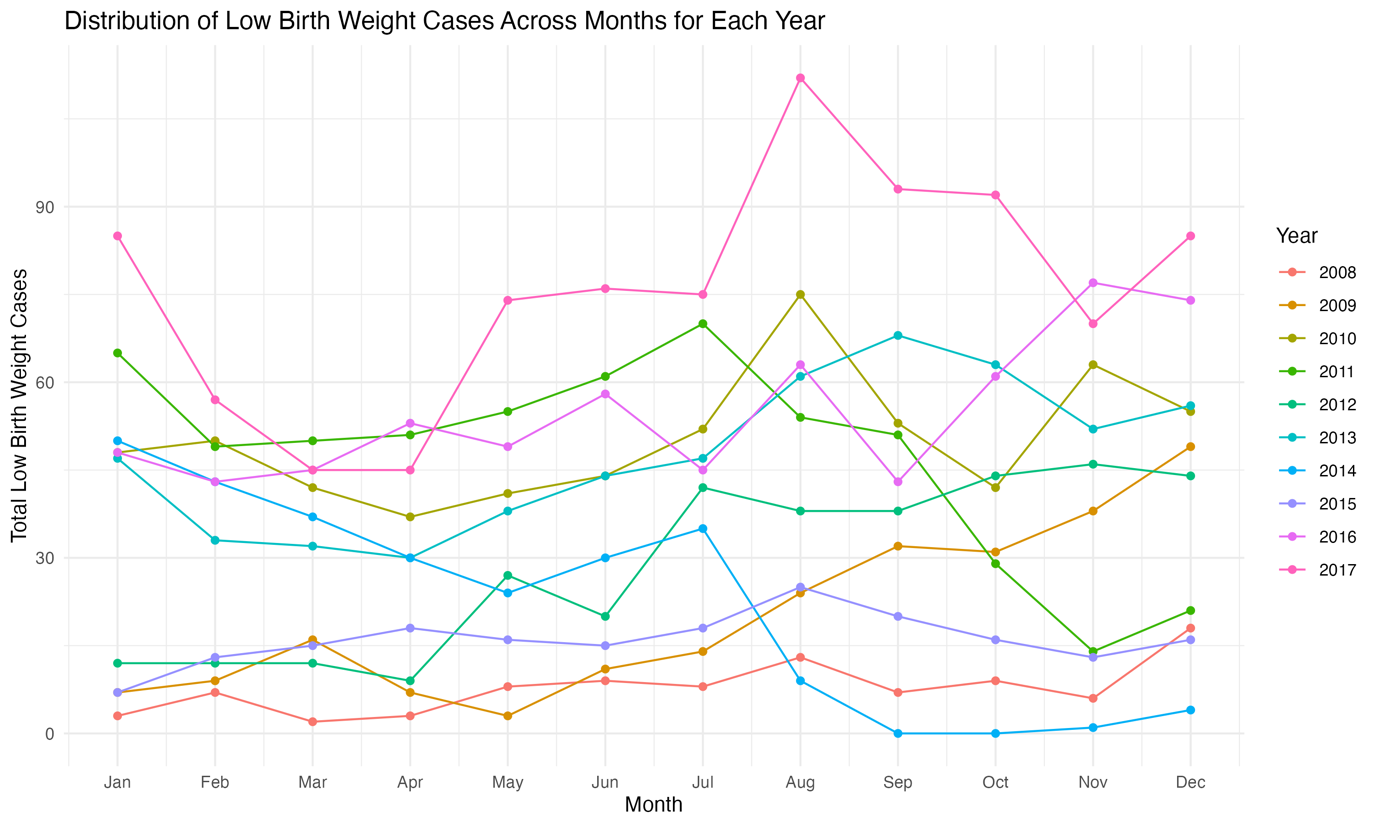


**Figure S1 Monthly distribution and patterns of LBW cases from 2008 to 2017.**

**Table S2 Cumulative and province-specific risk estimates of low birth weight (LBW) associated with heat index exposure.**

|  | **RR at extreme cold (1^st^ percentile)** | **RR at moderate cold (10^th^ percentile)** | **RR at moderate heat (90^th^ percentile)** | **RR at extreme heat (99^th^ percentile)** |
| --- | --- | --- | --- | --- |
| Cumulative | 0.27 (0.00, 63.06) | 0.32 (0.01, 7.79) | 5.58 (0.32, 97.31) | 8.55 (0.23, 314.53) |
| Baluchistan | 0.29 (0.00, 24.64) | 0.29 (0.00, 24.64) | 7.02 (0.23, 211.53) | 9.27 (0.18, 472.40) |
| GB | -- | -- | -- | -- |
| KPK | 0.42 (0.00, 84.30) | 0.44 (0.01, 23.29) | 9.46 (0.25, 355.22) | 12.08 (0.21, 685.18) |
| Punjab | 0.24 (0.00, 80.78) | 0.28 (0.00, 20.85) | 6.24 (0.22, 173.60) | 8.43 (0.16, 433.89) |
| Sindh | 0.15 (0.00, 51.75) | 0.19 (0.00, 12.37) | 4.32 (0.24, 78.62) | 5.40 (0.18, 161.76) |

**Table S3 Sensitivity analysis of model configurations assessing the relationship between temperature extremes and low birth weight (LBW).**

Relative risk (RR) estimates and 95% confidence intervals (CI) are shown for the 1st percentile (cold effect) and 99th percentile (heat effect) of the heat index, along with the corresponding QAIC for model fit. GLM models were tested with varying degrees of freedom (df) for long-term trend adjustment and maximum lag periods (lag = 7–9 months), with knots placed at the 50th percentile of the heat index. Additional specifications included natural spline functions and mixed-effects models (GLMMs) with random intercepts and/or zero inflation.

| **Model parameters** | **QAIC** | **1^st^ Percentile cold effects (95% CI)** | **99^th^ Percentile heat effects (95% CI)** |
| --- | --- | --- | --- |
| ***GLM Models: Sensitivity Analysis of Degrees of Freedom and Lag Periods*** | | | |
| df = 3, lag = 7 | 1278.92 | 0.07 (0.00, 3.80) | 8.72 (1.33, 57.11) |
| df = 4, lag = 7 | 1248.08 | 0.32 (0.00, 23.35) | 2.55 (0.34, 19.42) |
| df = 5, lag = 7 | 1156.23 | 2.09 (0.02, 221.82) | 1.07 (0.12, 9.67) |
| df = 6, lag = 7 | 1203.19 | 0.61 (0.01, 69.82) | 1.76 (0.18, 16.82) |
| df = 7, lag = 7 | 1074.63 | 9.30 (0.08, 1051.84) | 0.78 (0.09, 7.17) |
| df = 8, lag = 7 | 1121.41 | 1.90 (0.02, 230.13) | 1.90 (0.18, 20.05) |
| df = 9, lag = 7 | 1055.53 | 3.68 (0.03, 438.43) | 1.69 (0.16, 18.23) |
| df = 10, lag = 7 | 1050.00 | 2.62 (0.02, 320.77) | 2.00 (0.17, 22.87) |
| df = 15, lag = 7 | 982.27 | 0.53 (0.00, 68.61) | 3.83 (0.22, 66.53) |
| df = 20, lag = 7 | 996.10 | 0.56 (0.00, 97.29) | 4.04 (0.19, 85.82) |
| df = 30, lag = 7 | 1039.67 | 0.54 (0.00, 109.49) | 5.25 (0.22, 123.19) |
| df = 3, lag = 8 | 1276.26 | 0.06 (0.00, 5.68) | 10.66 (1.36, 83.88) |
| df = 4, lag = 8 | 1247.34 | 0.17 (0.00, 21.65) | 2.71 (0.29, 25.22) |
| df = 5, lag = 8 | 1155.51 | 1.10 (0.00, 270.65) | 0.87 (0.08, 10.03) |
| df = 6, lag = 8 | 1203.50 | 0.29 (0.00, 68.28) | 1.52 (0.12, 18.43) |
| df = 7, lag = 8 | 1074.47 | 5.86 (0.03, 1302.23) | 0.73 (0.06, 8.42) |
| df = 8, lag = 8 | 1120.60 | 1.17 (0.01, 242.89) | 2.10 (0.15, 28.78) |
| df = 9, lag = 8 | 1054.48 | 2.66 (0.1, 550.70) | 2.07 (0.14, 29.76) |
| df = 10, lag = 8 | 1050.06 | 2.03 (0.01, 440.30) | 2.24 (0.14, 35.70) |
| df = 15, lag = 8 | 981.43 | 0.34 (0.00, 58.55) | 4.99 (0.20, 122.32) |
| df = 20, lag = 8 | 995.16 | 0.36 (0.00, 86.30) | 5.17 (0.17, 153.25) |
| df = 30, lag = 8 | 1038.34 | 0.38 (0.00, 101.56) | 7.53 (0.22, 261.80) |
| df = 3, lag = 9 | 1270.11 | 0.05 (0.00, 8.61) | 13.39 (1.38, 130.26) |
| df = 4, lag = 9 | 1243.66 | 0.09 (0.00, 20.01) | 3.03 (0.26, 35.57) |
| df = 5, lag = 9 | 1154.29 | 0.31 (0.00, 178.79) | 0.75 (0.05, 11.14) |
| df = 6, lag = 9 | 1202.05 | 0.09 (0.00, 40.69) | 1.35 (0.08, 21.71) |
| df = 7, lag = 9 | 1070.43 | 2.61 (0.01, 1050.22) | 0.79 (0.05, 11.72) |
| df = 8, lag = 9 | 1115.59 | 0.59 (0.00, 191.40) | 2.67 (0.14, 49.98) |
| df = 9, lag = 9 | 1048.14 | 1.91 (0.00, 568.66) | 3.65 (0.18, 73.22) |
| df = 10, lag = 9 | 1045.51 | 1.26 (0.00, 395.63) | 3.60 (0.16, 83.48) |
| df = 15, lag = 9 | 976.37 | 0.27 (0.00, 63.07) | 8.54 (0.23, 314.53) |
| df = 20, lag = 9 | 989.89 | 0.28 (0.00, 90.80) | 8.04 (0.18, 353.62) |
| df = 30, lag = 9 | 1029.51 | 0.31 (0.00, 105.21) | 14.93 (0.26, 841.95) |
| ***GLM Models: Natural Splines with Specified Knots*** | | | |
| lag 9, ns = 3 | 976.37 | 0.27 (0.00, 63.07) | 8.54 (0.23, 314.53) |
| lag 9, ns = 4 | 989.02 | 0.27 (0.00, 65.15) | 8.76 (0.23, 336.61) |
| ***GLMM Models: Random Intercepts and/or Zero Inflation*** | | | |
| glmmTMB (*random intercepts and zero inflation)* | 2043.40* | 210.68 (16.17, 2745.49) | 3.55 (0.49, 25.76) |
| glmmTMB *(zero inflation)* | 2019.33* | 0.37 (0.01, 25.61) | 11.00 (0.51, 238.29) |

***AIC is estimated for glmmTMB models**
